# Pitfalls of assessment of autonomic function by heart rate variability

**DOI:** 10.1186/s40101-019-0193-2

**Published:** 2019-03-13

**Authors:** Junichiro Hayano, Emi Yuda

**Affiliations:** Department of Medical Education, Nagoya City University Graduate School of Medical Sciences, 1 Kawasumi Mizuho-cho Mizuho-ku, Nagoya, 467-8602 Japan

**Keywords:** Heart rate variability, Autonomic nervous system, Respiratory sinus arrhythmia, Cardiorespiratory coupling, Spectral analysis

## Abstract

Although analysis of heart rate variability is widely used for the assessment of autonomic function, its fundamental framework linking low-frequency and high-frequency components of heart rate variability with sympathetic and parasympathetic autonomic divisions has developed in the 1980s. This simplified framework is no longer able to deal with much evidence about heart rate variability accumulated over the past half-century. This review addresses the pitfalls caused by the old framework and discusses the points that need attention in autonomic assessment by heart rate variability.

## Background

Analysis of heart rate variability (HRV) is widely used as a standard method for assessing autonomic nervous functions. Particularly, low-frequency (LF, 0.04–0.15 Hz) and high-frequency (HF, 0.15–0.4 Hz) spectral components of HRV are used as the separate metrics of sympathetic and vagal (parasympathetic) functions [1, 2]. The association between HRV and autonomic function was first reported in the 1970s [3–6], and the currently prevailing frameworks of interpreting the HRV frequency components (LF and HF) in relation to the autonomic divisions were established in the 1980s [7–9]. Over the next half-century, more than 28,000 HRV studies were published, including a lot of evidence indicating important limitations of HRV frequency components as autonomic indices [10–12]. However, it does not seem that enough attention has been paid to such alarming evidence even by recent studies using HRV.

Analysis of HRV frequency components (LF and HF) provides a powerful tool for evaluating autonomic nervous function by simply recording an electrocardiogram (ECG). Furthermore, devices that automatically analyze HRV from ECGs, and also from pulse waves, are widely used in recent years. As a result, using the autonomic nervous index output there without criticism raises the risk of leading a wrong conclusion or a judgment that may harm health. Considering the recent knowledge of the mechanisms that generate HRV, the framework directly associating the HRV frequency components with the divisions of the autonomic nervous system (sympathetic and parasympathetic) has become too simplistic and it may be the time of reconstruction. This review will discuss the pitfalls where studies using HRV for autonomic assessment may fall based on the accumulated evidence of HRV.

## Main text

### General problems in the assessment of autonomic functions by HRV

#### Locality of autonomic neural regulations

The problem often seen in the studies of autonomic nerve function by HRV is interpretation as if HRV reflects the autonomic state of the whole body. The signals generating HRV are basically originated in the brain and mediated through the sympathetic and vagal nerves that innervate the sinoatrial node [13, 14]. Thus, the autonomic functions reflected in HRV are those regulating the pace-making function of the sinoatrial node. The autonomic nervous functions of other organ systems cannot be known from HRV. For example, food intake enhances parasympathetic (vagal) activity to stimulate digestive functions but it suppresses vagal activity to the heart to increase heart rate to a level that meets circulatory demand for digestion and absorption [15]. Although sympathetic regulation may parallel among cardiac, renal, and muscle sympathetic nerves [16, 17], parasympathetic regulation is by function and therefore HRV cannot be used for estimating parasympathetic regulation other than that for cardiac pace-making function.

#### Short-term and long-term HRV analyses

Analysis of HRV is classified into two categories by the length of data recording [2]. Short-term HRV is typically calculated over 5 min, and the magnitude of frequency components obtained by spectral analysis is used for autonomic function assessment. Long-term HRV is computed over nominal 24 h and time-domain [18] and frequency-domain [19] indices, nonlinear indices [20–23], and indices obtained by signal averaging [24–26] are used mainly for mortality risk prediction.

However, there is a more important difference between short-term and long-term HRV than the length of the data. For short-term HRV, data are usually recorded under monitoring while keeping subjects and measurement environment in specified conditions, while long-term HRV data are usually recorded by wearable sensors in subjects who are freely moving under daily activities. This difference is important for the interpretation of long-term HRV and the mechanisms by which it relates to clinical risks such as mortality.

**Fig. 1:**
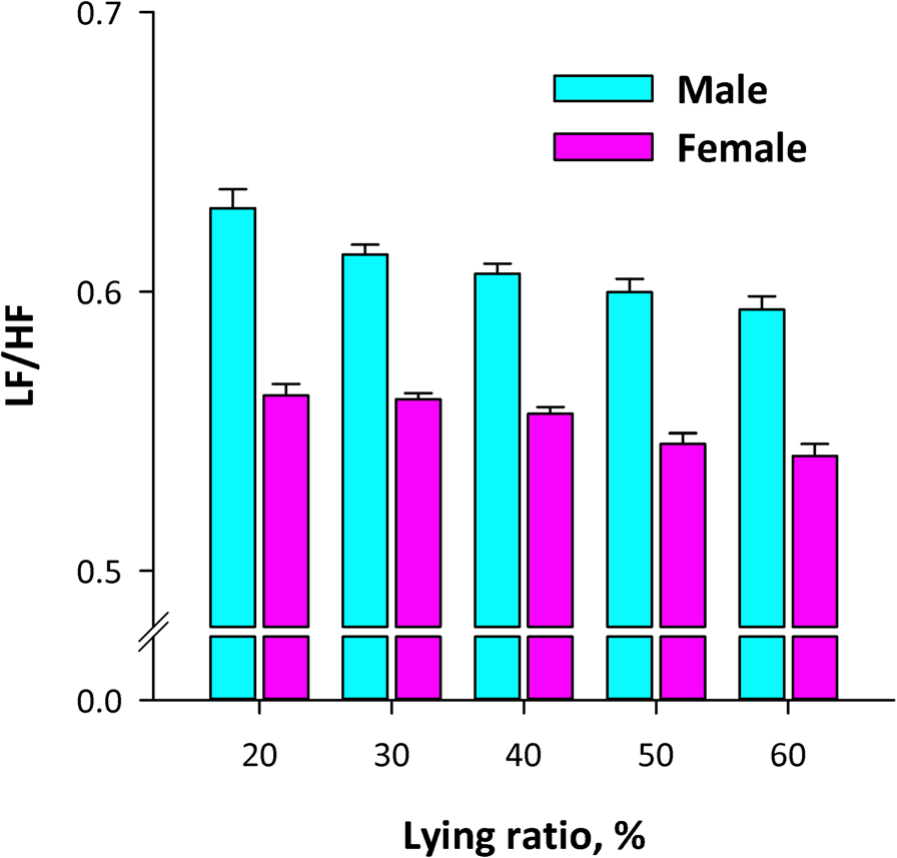
Association between LF/HF and lying ratio during 24-h monitoring in 18,944 men and 23,539 women who underwent 24-h Holter ECG monitoring with tri-axial accelerogram to assess physical activity and body position. Data were obtained from 24-h long-term HRV database of the Allostatic State Mapping by Ambulatory ECG Repository (ALLSTAR) project. (Revised figure in reference [31])

For example, decreased 24-h standard deviation of normal-to-normal R-R interval (SDNN), representative long-term time-domain HRV, is an increased mortality risk after acute myocardial infarction [18, 20]. This relationship is often described as cardiac vagal dysfunction to increase the vulnerability of the heart to ventricular tachycardia and fibrillation, but the autonomic nervous function may not be a sole determinant of 24-h SDNN. In a recent study using the big data of simultaneously recorded 24-h ECG and tri-axial accelerograms, we found that the major determinant of 24-h SDNN is day/night difference in R-R interval that was associated with the level of daytime physical activity [27]. This suggests that the association between reduced SDNN and adverse prognosis may be partly due to health conditions that restrict daily physical activity. Also, LF-to-HF power ratio (LF/HF) has been widely used as autonomic index, and it is well known that LF/HF increases with standing [7, 8, 28]. The major studies of the association between long-term HRV and disease prognosis reported that a decrease in LF/HF is an increased mortality risk [20, 22, 29]. In recent studies using big data of long-term HRV and accelerogram, we observed that the LF/HF reduces with the increasing lying period during 24 h (Fig. 1) [30, 31]. Decreased LF/HF in patients with adverse prognosis may reflect their daily life spending a long time in lying position due to health problems.

These indicate that long-term HRV is affected by physical activity and posture during the recording period and that the relationships with autonomic functions known for short-term HRV cannot be extended to the interpretation of long-term HRV. To use long-term HRV to evaluate autonomic nervous function during daily life, it is necessary to monitor at least physical activity and posture and analyze HRV in relation to them.

### Separation of autonomic divisions by frequency

#### Why 0.15 Hz?

LF and HF component of HRV is divided at 0.15 Hz. Classification at this frequency was proposed by Sayers [5] and supported by animal experiments [32, 33]. Among those, a study by Berger et al. [33] performed the transfer function analysis of cardiac sinus rate response to the broad-frequency electric stimulation of the sympathetic and vagal nerves in dogs. They found that the sinus node responds as a low-pass filter to fluctuations in either sympathetic or vagal tone, while the filter for sympathetic stimulations has a lower corner frequency (0.15 Hz) than for vagal stimulations (> 0.5 Hz).

It is believed that the difference in frequency characteristics between sympathetic and vagal heart rate controls arises from the difference in the signaling mechanisms between beta-adrenergic and cholinergic receptors. Changes in sympathetic activity cause the effects through several phosphorylating enzymatic processes in the intracellular signal transduction mechanisms existing downstream of beta-adrenergic receptors. Consequently, the sympathetic modulation of heart rate cannot transfer fast fluctuations (> 0.15 Hz). On the other hand, changes in vagal activity cause the effects simply by the conformation change of the membrane potassium channels with Ach. Consequently, the vagal modulation of heart rate can transfer fluctuations up to a higher frequency range. The cutoff frequency of 0.15 Hz, however, is the value obtained from dogs, and there is no convincing evidence as to whether the value applies to humans of all ages uner all conditions.

#### Sinus arrhythmias constituting frequency component of HRV

The message brought about by studies showing the difference in frequency characteristics between sympathetic and vagal heart rate regulations is often over-interpreted. It is not saying that the magnitude of HF component of HRV reflects cardiac vagal function but is only saying that when heart rate fluctuations mediated by autonomic nerves are observed in HF band (> 0.15 Hz), it is mediated by the cardiac vagus. Indeed, even if HRV in HF band decreased or disappeared, it may not necessarily mean a withdrawal of cardiac vagal activity or cardiac vagal dysfunction. This could happen when the respiration frequency is out of the HF band, such as slow breathing < 0.15 Hz (9 breath/min) like during deep breathing, or fast breathing > 0.4 Hz (24 breath/min) during exercise and in children. Also, even when heart rate fluctuation is observed in HF band (> 0.15 Hz), if it is not mediated by autonomic nerves (as discussed in the next section), the framework of frequency separation of the autonomic heart rate regulation cannot be used.

Considering the knowledge of the mechanisms that generate HRV, the framework to directly associate the HRV frequency with the divisions of the autonomic nervous system (sympathetic and vagal) is too simplistic and already classical. HRV should be described at least dividing it into individual fluctuations from the corresponding physiological mechanisms. It is well known that there are two major components in short-term HRV; respiratory sinus arrhythmia (RSA) and a fluctuation at ~ 0.1 Hz called Mayer wave sinus arrhythmia (MWSA) [2, 7, 8]. The autonomic mechanisms of RSA are discussed in later sections. The mechanism mediating the heart rate fluctuation at ~ 0.1 Hz is believed to be the baroreceptor reflex cardiovascular regulation system, by which the fluctuation of arterial blood pressure at ~ 0.1 Hz that are known as Mayer wave [34] is reflected in the fluctuation of heart rate [12, 35, 36]. Therefore, we named this HRV component “Mayer wave sinus arrhythmia [15, 37].”

As mentioned above, when respiration frequency is < 0.15 Hz, RSA becomes a part of the LF component and the association between HF component and cardiac vagal function is lost. Additionally, when consciously breathing at ~ 0.1 Hz, it may cause resonance among respiration, heart rate, and blood pressure through the baroreceptor reflex mechanism, by which RSA and MWSA are merged into a large single oscillation at ~ 0.1 Hz [38, 39].

#### Non-vagal HRV component in HF band

Even if HRV is observed in the HF band, it may not necessarily be mediated by autonomic nerves. This phenomenon has been known for a long time and has been reported in different terms including complex HRV [40, 41], sinus node alternans [42], erratic heart rate [43, 44], and heart rate fragmentation (HRF; we will use this term in this review) [23, 45, 46]. This is a type of sinoatrial instability characterized by frequent (sometimes every beat) appearance of peak and valley in R-R interval time series despite that ECG shows sinus rhythm. By this phenomenon, switching between increases and decreases in heart rate occurs more frequently than those expected for that mediated by the vagus.

To quantify the level of HRF, Costa et al. [45, 46] developed a set of metrics including the percentage of the inflection point (PIP) and symbol dynamics [45]. Using these measures, they demonstrated that the level of HRF increases with advancing age and in patients with coronary artery disease [45] and that its increase is an increased risk of cardiovascular events and death [23]. Because the existence of this phenomenon appears as increases in the conventional HRV metrics such as the percentage of pairs of adjacent normal-to-normal R-R intervals differing by > 50 ms (pNN50), the square root of the mean of squared differences between adjacent normal-to-normal R-R intervals (RMSSD), and HF power, it may confound their associations with cardiac vagal function and with disease prognosis. In fact, this phenomenon is considered a potential cause of lesser prognostic relevance of the HF component than that of other HRV frequency components such as very-low-frequency (0.0033–0.04 Hz) and LF components [43]. Although the exact mechanisms for this phenomenon are unclear, alternative pacemaker shift within the sinus node caused by sinoatrial degeneration or disorganization may be a potential mechanism.

### RSA and cardiac vagal function

#### Non-vagal factors influencing RSA

Although the association between HF component of short-term HRV and cardiac vagal function depends on the neural mechanisms of RSA [47, 48], many factors are known to influence the magnitude of RSA besides the cardiac vagal function. An increase in respiratory frequency decreases the amplitude of RSA (the range of excursion of R-R interval with respiration) [49–51]. Also, an increase in tidal volume increases RSA amplitude [49–53]. These effects of respiratory parameters on the RSA operate independently of the level of cardiac vagal activity [54]. Therefore, when using RSA amplitude to evaluate cardiac vagal function, it is desirable to control respiration parameters in experimental protocol or to adjust their effects statistically.

Also, RSA amplitude may be affected by sympathetic nervous activity. Even though the sympathetic nervous system cannot transfer HRV > 0.15 Hz [33], it may restrict the magnitude of cardiac vagal modulations of heart rate [55, 56]. Taylor et al. [55] observed that beta-adrenergic blockade increases RSA amplitude over a wide frequency range including > 0.15 Hz and concluded that cardiac sympathetic outflow reduces heart rate oscillations at all frequencies including RSA.

#### Neural mechanisms of RSA

Despite that RSA amplitude is widely used to estimate cardiac vagal activity that is controlling the level of heart rate, RSA amplitude and the level of heart rate are known to be controlled by different groups of vagal motor neurons [57]. Preganglionic cardiac vagal motor neurons are known to locate separately in the dorsal motor nucleus of the vagus (DVN) and in the nucleus ambiguous (nA) [57]. In cats and rats, C-fiber cardiac preganglionic neurons in the DVN shows regular ongoing activity that is unaffected by respiratory rhythm, while those in the nA fire with respiratory rhythm [58]. During embryonic development, the cardiac preganglionic neurons forming nA migrate ventrolaterally from a dorsomedial position, possibly of the DVN [59]. In axolotl, this neuron relocation occurs with the metamorphosis of the onset of air-breathing [60]. These suggest that during the evolution of air-breathing vertebrate, the nA was developed to “generate” RSA in addition to the DVN to control the level of heart rate.

#### Biological function of RSA

**Fig. 2:**
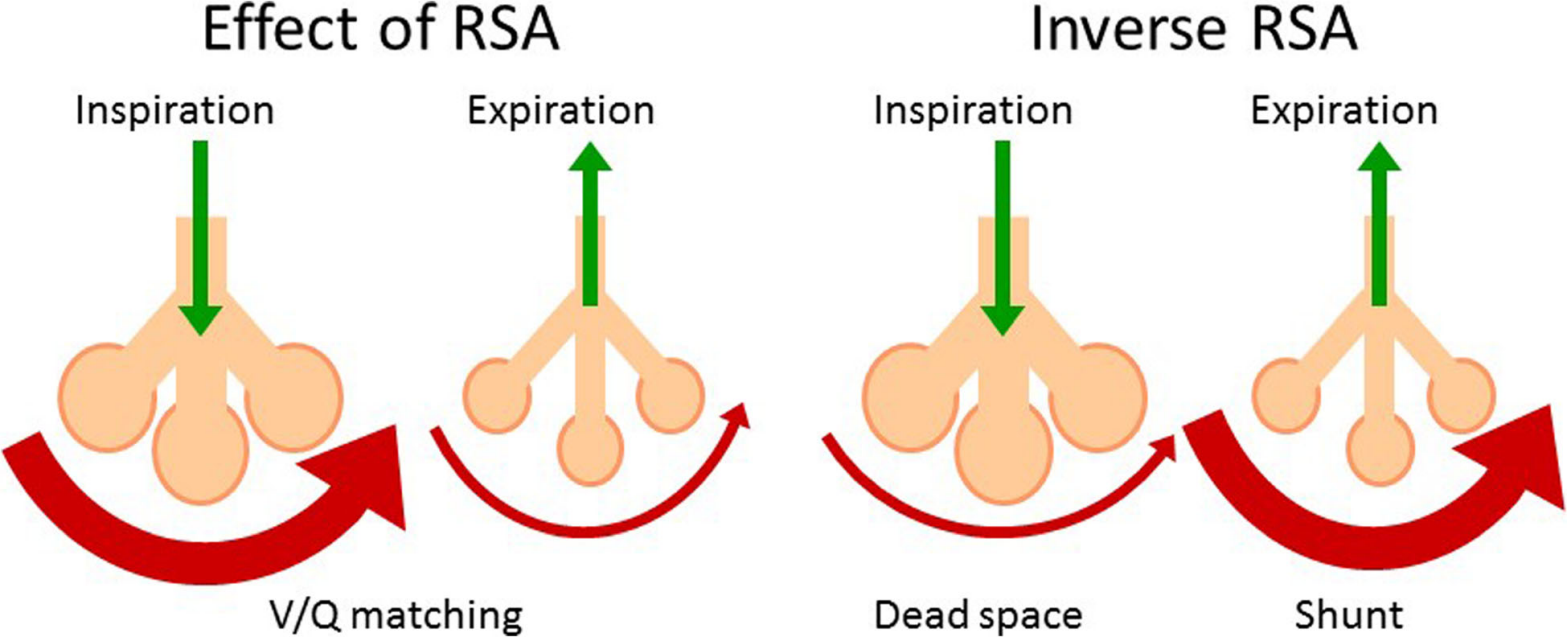
Schema of the effects of physiological RSA and its inversion (inverse RSA) on the relationship between alveolar gas volume and capillary blood flow during inspiration and expiration. Horizontal red bows and vertical green arrows indicate the volume of blood flow and the direction of gas flow, respectively. Physiological RSA improves respiratory gas exchange efficiency through matching between alveolar ventilation and capillary perfusion throughout the respiratory cycle, while the inversion of the relationship (inverse RSA) results in increased alveolar dead space (wasted ventilation) and increased intrapulmonary shunt. (Revised figure in reference [61])

Beside its relationship to cardiac vagal function, RSA itself is recognized as a physiological mechanism to ensure optimal ventilation-perfusion matching within the lungs of air-breathing mammals [11, 57, 61–63]. A similar matching of the counter-perfusions with water and blood of the gills is also known in aquatic animals without the nA, but this matching is accomplished simply by adjusting the level of heart rate with ventilatory gill movements [64]. In air-breathing animals with reciprocal respiration, the presence of RSA may improve pulmonary gas exchange efficiency by matching the timing of alveolar air volume and alveolar perfusion within each respiratory cycle (Fig. 2) [61]. Because this mechanism could reduce the intrapulmonary shunt ratio by reducing ineffective heartbeats when the alveoli are contracting and it could also suppress the functional dead space by reducing wasted inspiration when alveolar perfusion is small, RSA seems a biological function to save the energy for circulation and respiration at rest—the period when the energy saving of the pulmonary circulation is more important to survive against hunger than responding to the demand for systemic circulation [11].

#### Possible reason why RSA is associated with cardiac vagal activity

If RSA and heart rate are regulated by different vagal motor neurons, direct relationship may not be expected between the magnitude of RSA and cardiac vagal activity to control heart rate. In fact, Goldberger et al. [65, 66] demonstrated the evidence of dissociation between RSA magnitude and cardiac vagal activity. They observed that bradycardia caused by cardiac vagal activation through baroreceptor stimulation with phenylephrine that increases arterial blood pressure is accompanied by a paradoxical decrease in RSA amplitude. This indicates that the degree of respiratory modulation of cardiac vagal outflow is not always parallel to cardiac vagal tone (mean activity level).

#### RSA as an indicator of intrinsic resting function

**Fig. 3:**
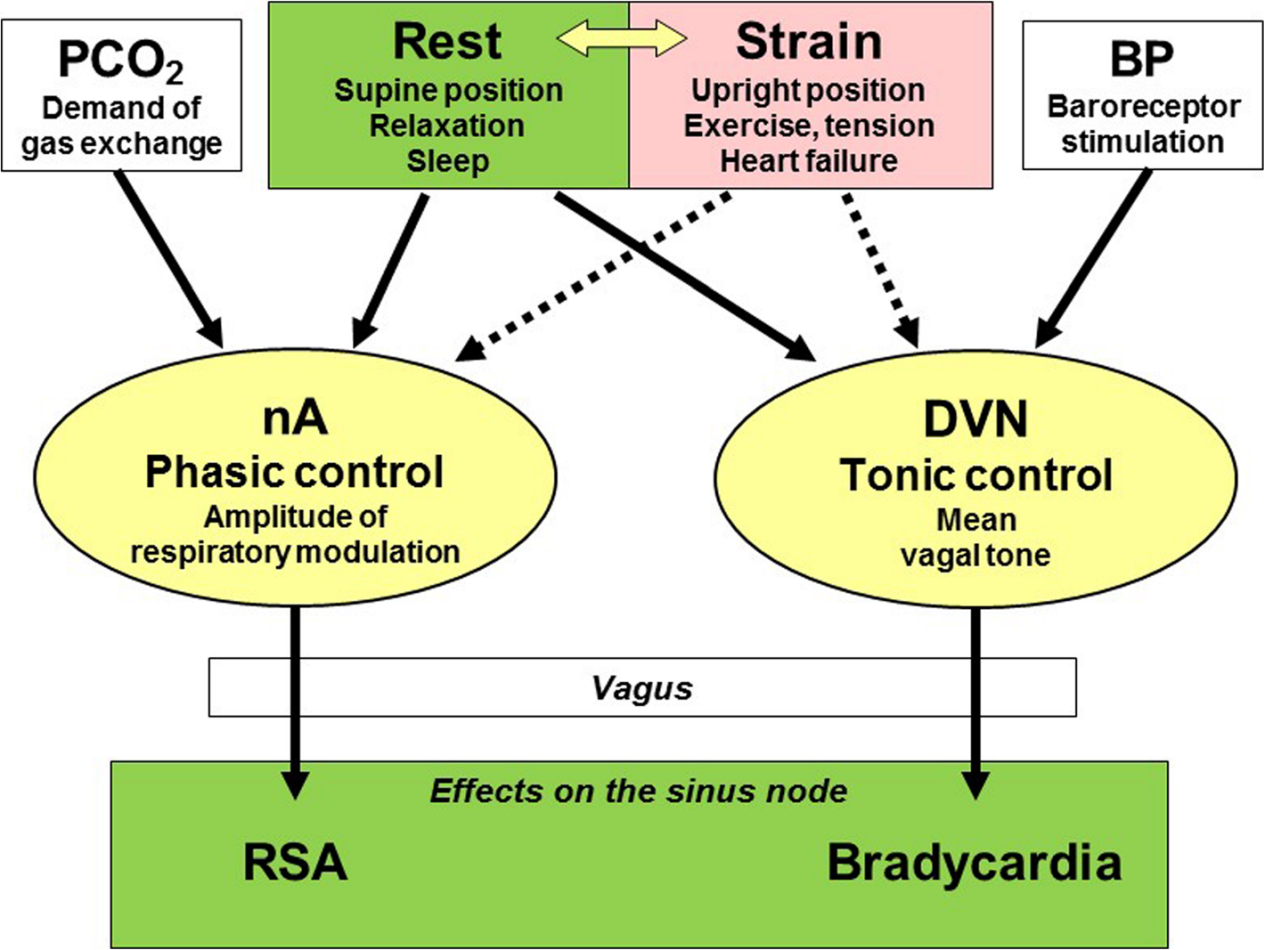
Model of central regulations RSA and the level of heart rate by preganglionic cardiac vagal motor neurons. RSA is generated by the phasic control system located in the nucleus ambiguous (nA) that regulates the amplitude of respiratory modulation of cardiac vagal outflow, while bradycardia is derived by the tonic control system located in the dorsal motor nucleus of the vagus (DVN) that regulates average cardiac vagal tone. These systems work independently of HRV of each other and are stimulated (solid arrows) or inhibited (broken arrows) by different kinds of inputs. However, both systems appear to work in parallel and to link with each other, whenever the cardiac autonomic state changes along with the rest-strain axis. (Revised figure in reference [61])

Nevertheless, why is the RSA amplitude widely used as an indicator of cardiac vagal activity? This is probably because RSA is an intrinsic resting function that appears when an individual is about to rest. In such situations, the nA and DVN are expected to work cooperatively for the common purpose—energy saving (Fig. 3). This apparently indicates that RSA amplitude is a limited indicator of cardiac vagal activity, but at the same time, it suggests that RSA amplitude may be a useful indicator of resting function. The latter assumption seems supported by much evidence about the factors affecting RSA amplitude; i.e., RSA increases with supine posture [7], relaxation [67], and sleep [68], decreases with standing [7, 8, 28], physical exercise [69–71], mental stress [72], cardiovascular stress including baroreceptor stimulation [65, 66], advancing age [73], and compromised cardiac function [37, 74].

### LF component and sympathetic function

Although the power of the LF component and its derived indices (normalized LF power and LF/HF) have been widely used as an indicator of sympathetic functions (activity, predominance, sympatho-vagal balance, etc.), the validity of this use has been denied by a lot of evidence accumulated by numerous studies [12]. Unlike the studies specific to RSA in the HF component, this conclusion does not change, even if the discussion is specified to MWSA. Earlier studies proposed the LF as sympathetic component [8], but the power of LF spectrum is not eliminated completely by beta-adrenergic blockade and rather largely reduced by sinoatrial parasympathectomy [75]. Although the increase in LF power with standing or head-up tilting is often cited as the evidence for its association with sympathetic activity, postural increase in LF power is observed only in one-third of healthy subjects, no significant change in other third, and rather decreases in the remaining third [28]. While LF/HF increases with standing, this is mainly due to the postural decrease in HF power. Finally, LF power is not related to cardiac sympathetic innervation quantified by positron emission tomographic neuroimaging [36, 76]. There is no longer a convincing physiological basis justifying the assessment of cardiac sympathetic nervous functions whether by absolute or normalized LF power or LF/HF.

## Conclusions

This paper described the major pitfalls of the assessment of autonomic function by HRV. Overall, HRV tends to be used beyond the limit of its capacity as a quantitative measure of autonomic function. Particularly, the framework to associate the HRV frequency components (LF and HF) with the divisions of the autonomic nervous system (sympathetic and parasympathetic) is already too simplistic, and it is the major cause of the pitfalls.

On the other hand, even if HRV is a limited measure of autonomic functions, it is still a fact that HRV is there. Except for some exception such as HRF, HRV is thought to originate from the brain and to transfer to the heart through the autonomic nervous system. Like the radio that extracts information from radio waves modulated by the broadcasting station, HRV analysis can intercept the information for circulatory regulation generated by the brain. We will be able to expect the continued development of HRV researches by aiming for a faithful and unbiased extraction of the information contained in HRV beyond the classic framework of LF and HF.

## Abbreviations

Ach: Acetylcholine
DVN: Dorsal motor nucleus of the vagus
ECG: Electrocardiogram
HF: High frequency
HRF: Heart rate fragmentation
HRV: Heart rate variability
LF: Low frequency
LF/HF: LF-to-HF ratio
MWSA: Mayer wave sinus arrhythmia
nA: Nucleus ambiguous
PIP: Percentage of inflection point
pNN50: Percentage of pairs of adjacent normal-to-normal R-R intervals differing by > 50 ms
RMSSD: Square root of the mean of squared differences between adjacent normal-to-normal R-R intervals
RSA: Respiratory sinus arrhythmia
SDNN: Standard deviation of normal-to-normal R-R interval

## References

[1] Appel ML, Berger RD, Saul JP, Smith JM, Cohen RJ (1989). Beat to beat variability in cardiovascular variables: noise or music?. J Am Coll Cardiol.

[2] Camm AJ, Malik M, Bigger JT, Breithardt G, Cerutti S, Cohen RJ, Coumel P, Fallen EL, Kleiger RE, Lombardi F, Malliani A, Moss AJ, Rottman JN, Schmidt G, Schwartz PJ, Singer DH, Task force of the European Society of Cardiology and the north American Society of Pacing and Electrophysiology (1996). Heart rate variability: standards of measurement, physiological interpretation and clinical use. Circulation..

[3] Katona PG, Poitras JW, Barnett GO, Terry BS (1970). Cardiac vagal efferent activity and heart period in the carotid sinus reflex. Am J Phys.

[4] Hyndman BW, Kitney RI, Sayers BM (1971). Spontaneous rhythms in physiological control systems. Nature..

[5] Sayers BM (1973). Analysis of heart rate variability. Ergonomics..

[6] Hyndman BW, Gregory JR (1975). Spectral analysis of sinus arrhythmia during mental loading. Ergonomics..

[7] Pomeranz B, Macaulay RJ, Caudill MA, Kutz I, Adam D, Gordon D, Kilborn KM, Barger AC, Shannon DC, Cohen RJ (1985). Assessment of autonomic function in humans by heart rate spectral analysis. Am J Phys.

[8] Pagani M, Lombardi F, Guzzetti S, Rimoldi O, Furlan R, Pizzinelli P, Sandrone G, Malfatto G, Dell'Orto S, Piccaluga E, Turiel M, Baselli G, Cerutti S, Malliani A (1986). Power spectral analysis of heart rate and arterial pressure variabilities as a marker of sympatho-vagal interaction in man and conscious dog. Circ Res.

[9] Billman GE (2011). Heart rate variability - a historical perspective. Front Physiol.

[10] Berntson GG, Bigger JT, Eckberg DL, Grossman P, Kaufmann PG, Malik M, Nagaraja HN, Porges SW, Saul JP, Stone PH, Van der Molen MW (1997). Heart rate variability: origins, methods, and interpretive caveats. Psychophysiology..

[11] Hayano J, Yasuma F (2003). Hypothesis: respiratory sinus arrhythmia is an intrinsic resting function of cardiopulmonary system. Cardiovasc Res.

[12] Billman GE (2013). The LF/HF ratio does not accurately measure cardiac sympatho-vagal balance. Front Physiol.

[13] Sands KEF, Appel ML, Lilly LS, Schoen FJ, Mudge GH, Cohen RJ (1989). Power spectrum analysis of heart rate variability in human cardiac transplant recipients. Circulation..

[14] Hayano J, Sakakibara Y, Yamada A, Yamada M, Mukai S, Fujinami T, Yokoyama K, Watanabe Y, Takata K (1991). Accuracy of assessment of cardiac vagal tone by heart rate variability in normal subjects. Am J Cardiol.

[15] Hayano J, Sakakibara Y, Yamada M, Kamiya T, Fujinami T, Yokoyama K, Watanabe Y, Takata K (1990). Diurnal variations in vagal and sympathetic cardiac control. Am J Phys.

[16] Kamiya A, Kawada T, Yamamoto K, Michikami D, Ariumi H, Miyamoto T, Shimizu S, Uemura K, Aiba T, Sunagawa K, Sugimachi M (2005). Dynamic and static baroreflex control of muscle sympathetic nerve activity (SNA) parallels that of renal and cardiac SNA during physiological change in pressure. Am J Phys.

[17] Kamiya A, Kawada T, Yamamoto K, Michikami D, Ariumi H, Miyamoto T, Uemura K, Sugimachi M, Sunagawa K (2005). Muscle sympathetic nerve activity averaged over 1 minute parallels renal and cardiac sympathetic nerve activity in response to a forced baroreceptor pressure change. Circulation..

[18] Kleiger RE, Miller JP, Bigger JT, Moss AJ, the Multicenter Post-Infarction Research G (1987). Decreased heart rate variability and its association with increased mortality after acute myocardial infarction. Am J Cardiol.

[19] Bigger JT, Fleiss JL, Steinman RC, Rolnitzky LM, Kleiger RE, Rottman JN (1992). Frequency domain measures of heart period variability and mortality after myocardial infarction. Circulation..

[20] Huikuri HV, Mäkikallio TH, Peng CK, Goldberger AL, Hintze U, Moller M, Grp DS (2000). Fractal correlation properties of R-R interval dynamics and mortality in patients with depressed left ventricular function after an acute myocardial infarction. Circulation..

[21] Kiyono K, Hayano J, Watanabe E, Struzik ZR, Yamamoto Y (2008). Non-Gaussian heart rate as an independent predictor of mortality in patients with chronic heart failure. Heart Rhythm.

[22] Suzuki M, Hiroshi T, Aoyama T, Tanaka M, Ishii H, Kisohara M, Iizuka N, Murohara T, Hayano J (2012). Nonlinear measures of heart rate variability and mortality risk in hemodialysis patients. Clin J Am Soc Nephrol.

[23] Costa MD, Redline S, Davis RB, Heckbert SR, Soliman EZ, Goldberger AL (2018). Heart rate fragmentation as a novel biomarker of adverse cardiovascular events: the multi-ethnic study of atherosclerosis. Front Physiol.

[24] Schmidt G, Malik M, Barthel P, Schneider R, Ulm K, Rolnitzky L, Camm AJ, Bigger JT, Schomig A (1999). Heart-rate turbulence after ventricular premature beats as a predictor of mortality after acute myocardial infarction. Lancet..

[25] Bauer A, Kantelhardt JW, Barthel P, Schneider R, Makikallio T, Ulm K, Hnatkova K, Schomig A, Huikuri H, Bunde A, Malik M, Schmidt G (2006). Deceleration capacity of heart rate as a predictor of mortality after myocardial infarction: cohort study. Lancet..

[26] Hayano J, Yasuma F, Watanabe E, Carney RM, Stein PK, Blumenthal JA, Arsenos P, Gatzoulis KA, Takahashi H, Ishii H, Kiyono K, Yamamoto Y, Yoshida Y, Yuda E, Kodama I (2017). Blunted cyclic variation of heart rate predicts mortality risk in post-myocardial infarction, end-stage renal disease, and chronic heart failure patients. Europace..

[27] Hayano J, Yuda E, Furukawa Y, Yoshida Y (2018). Association of 24-hour heart rate variability and daytime physical activity: ALLSTAR big data analysis. Int J Biosci Biochem Bioinformatics.

[28] Hayano J, Mukai S, Fukuta H, Sakata S, Ohte N, Kimura G (2001). Postural response of low-frequency component of heart rate variability is an increased risk for mortality in patients with coronary artery disease. Chest..

[29] La Rovere MT, Bigger JT, Marcus FI, Mortara A, Schwartz PJ, Investigators A (1998). Baroreflex sensitivity and heart-rate variability in prediction of total cardiac mortality after myocardial infarction. Lancet..

[30] Yoshida Y, Ogasawara H, Yuda E, Hayano J. What does LF/HF of heart rate variability in ambulatory ECG mean? Effect of time in lying position during monitoring. Eur Heart J. 2016;37(suppl):96-97.

[31] Yoshida Y, Furukawa Y, Ogasawara H, Yuda E, Hayano J (2016). Longer lying position causes lower LF/HF of heart rate variability during ambulatory monitoring.

[32] Akselrod S, Gordon D, Ubel FA, Shannon DC, Barger AC, Cohen RJ (1981). Power spectrum analysis of heart rate fluctuation: a quantitative probe of beat-to-beat cardiovascular control. Science..

[33] Berger RD, Saul JP, Cohen RJ (1989). Transfer function analysis of autonomic regulation. I. Canine atrial rate response. Am J Phys.

[34] Penaz J. Mayer waves: history and methodology. Automedica. 1978;2:135-141.

[35] Madwed JB, Albrecht P, Mark RG, Cohen RJ (1991). Low-frequency oscillation in arterial pressure and heart rate: a simple computer model. Am J Phys.

[36] Rahman F, Pechnik S, Gross D, Sewell L, Goldstein DS (2011). Low frequency power of heart rate variability reflects baroreflex function, not cardiac sympathetic innervation. Clin Auton Res.

[37] Hayano J, Sakakibara Y, Yamada M, Ohte N, Fujinami T, Yokoyama K, Watanabe Y, Takata K (1990). Decreased magnitude of heart rate spectral components in coronary artery disease. Its relation to angiographic severity. Circulation..

[38] Vaschillo EG, Vaschillo B, Lehrer PM (2006). Characteristics of resonance in heart rate variability stimulated by biofeedback. Appl Psychophysiol Biofeedback.

[39] Lehrer PM, Gevirtz R (2014). Heart rate variability biofeedback: how and why does it work?. Front Psychol.

[40] Woo MA, Stevenson WG, Moser DK, Middlekauff HR (1994). Complex heart rate variability and serum norepinephrine levels in patients with advanced heart failure. J Am Coll Cardiol.

[41] Woo MA, Stevenson WG, Moser DK, Trelease RB, Harper RM (1992). Patterns of beat-to-beat heart rate variability in advanced heart failure. Am Heart J.

[42] Binkley PF, Eaton GM, Nunziata E, Khot U, Cody RJ (1995). Heart rate alternans. Ann Intern Med.

[43] Stein PK, Domitrovich PP, Hui N, Rautaharju P, Gottdiener J (2005). Sometimes higher heart rate variability is not better heart rate variability: results of graphical and nonlinear analyses. J Cardiovasc Electrophysiol.

[44] Stein PK, Le Q, Domitrovich PP, Investigators C (2008). Development of more erratic heart rate patterns is associated with mortality post-myocardial infarction. J Electrocardiol.

[45] Costa MD, Davis RB, Goldberger AL (2017). Heart rate fragmentation: a symbolic dynamical approach. Front Physiol.

[46] Costa MD, Davis RB, Goldberger AL (2017). Heart rate fragmentation: a new approach to the analysis of cardiac interbeat interval dynamics. Front Physiol.

[47] Taylor EW (1994). The evolution of efferent vagal control of the heart in vertebrates. Cardioscience..

[48] Taylor EW, Hoar WS, Randall DJ, Farrell AP (1992). Nervous control of the heart and cardiorespiratory interactions. Fish Physiology vol XII B.

[49] Melcher A (1976). Respiratory sinus arrhythmia in man: a study in heart rate regulating mechanisms. Acta Physiol Scand.

[50] Hirsch JA, Bishop B (1981). Respiratory sinus arrhythmia in humans: how breathing pattern modulates heart rate. Am J Phys.

[51] Brown TE, Beightol LA, Koh J, Eckberg DL (1993). Important influence of respiration on human R-R interval power spectra is largely ignored. J Appl Physiol.

[52] Eckberg DL (1983). Human sinus arrhythmia as an index of vagal cardiac outflow. J Appl Physiol.

[53] Kollai M, Mizsei G (1990). Respiratory sinus arrhythmia is a limited measure of cardiac parasympathetic control in man. J Physiol (Lond).

[54] Hayano J, Mukai S, Sakakibara M, Okada A, Takata K, Fujinami T (1994). Effects of respiratory interval on vagal modulation of heart rate. Am J Phys.

[55] Taylor JA, Myers CW, Halliwill JR, Seidel H, Eckberg DL (2001). Sympathetic restraint of respiratory sinus arrhythmia: implications for vagal-cardiac tone assessment in humans. Am J Phys.

[56] Cohen MA, Taylor JA (2002). Short-term cardiovascular oscillations in man: measuring and modelling the physiologies. J Physiol.

[57] Taylor EW, Jordan D, Coote JH (1999). Central control of the cardiovascular and respiratory systems and their interactions in vertebrates. Physiol Rev.

[58] Jones JFX, Wang Y, Jordan D (1998). Activity of C-fiber cardiac vagal efferents in anaesthetized cats and rats. J Physiol (Lond).

[59] Windle WF (1933). Neurofibrillar development in the central nervous system of cat embryos between 8 and 12 mm long. J Comp Neurol.

[60] Taylor EW, Al-Ghamdi MS, Ihmied IH, Wang T, Abe AS (2001). The neuranatomical basis of central control of cardiorespiratory interactions in vertebrates. Exp Physiol.

[61] Hayano J, Yasuma F, Okada A, Mukai S, Fujinami T (1996). Respiratory sinus arrhythmia. A phenomenon improving pulmonary gas exchange and circulatory efficiency. Circulation..

[62] Yasuma F, Hayano J (2004). Respiratory sinus arrhythmia: why does the heartbeat synchronize with respiratory rhythm?. Chest..

[63] Ito S, Sasano H, Sasano N, Hayano J, Fisher JA, Katsuya H (2006). Vagal nerve activity contributes to improve the efficiency of pulmonary gas exchange in hypoxic humans. Exp Physiol.

[64] Satchell GH (1960). The reflex co-ordination of the heart beats with respiration in dogfish. J Exp Biol.

[65] Goldberger JJ, Ahmed MW, Parker MA, Kadish AH (1994). Dissociation of heart rate variability from parasympathetic tone. Am J Phys.

[66] Goldberger JJ, Challapalli S, Tung R, Parker MA, Kadish AH (2001). Relationship of heart rate variability to parasympathetic effect. Circulation..

[67] Sakakibara M, Takeuchi S, Hayano J (1994). Effect of relaxation training on cardiac parasympathetic tone. Psychophysiology..

[68] Bonnet MH, Arand DL (1997). Heart rate variability: sleep stage, time of night, and arousal influences. Electroencephalogr Clin Neurophysiol.

[69] Arai Y, Saul JP, Albrecht P, Hartley LH, Lilly LS, Cohen RJ, Colucci WS (1989). Modulation of cardiac autonomic activity during and immediately after exercise. Am J Phys.

[70] Yamamoto Y, Hughson RL, Peterson JC (1991). Autonomic control of heart rate during exercise studied by heart rate variability spectral analysis. J Appl Physiol.

[71] Taylor JA, Hayano J, Seals DR (1995). Lesser vagal withdrawal during isometric exercise with age. J Appl Physiol.

[72] Sakakibara M, Kanematsu T, Yasuma F, Hayano J (2008). Impact of real-world stress on cardiorespiratory resting function during sleep in daily life. Psychophysiology..

[73] Shannon DC, Carley DW, Benson H (1987). Aging of modulation of heart rate. Am J Phys.

[74] Saul JP, Arai Y, Berger RD, Lilly LS, Colucci WS, Cohen RJ (1988). Assessment of autonomic regulation in chronic congestive heart failure by heart rate spectral analysis. Am J Cardiol.

[75] Randall DC, Brown DR, Raisch RM, Yingling JD, Randall WC (1991). SA nodal parasympathectomy delineates autonomic control of heart rate power spectrum. Am J Phys.

[76] Moak JP, Goldstein DS, Eldadah BA, Saleem A, Holmes C, Pechnik S, Sharabi Y (2007). Supine low-frequency power of heart rate variability reflects baroreflex function, not cardiac sympathetic innervation. Heart Rhythm.

